# Neural-network-inspired federated coordination for noise-tolerant distributed optimization in cyber-physical demand-side management

**DOI:** 10.3389/fncom.2026.1895688

**Published:** 2026-07-23

**Authors:** Atef Gharbi, Ahmad Alshammari, Nasser Albalawi, Nadhir Ben Halima

**Affiliations:** 1 Department of Information Systems, Faculty of Computing and Information Technology, Northern Border University, Rafha, Saudi Arabia; 2 Department of Computer Sciences, Faculty of Computing and Information Technology, Northern Border University, Rafha, Saudi Arabia; 3 Department of Information Technology, Community College of Qatar, Doha, Qatar

**Keywords:** computational neuroscience, cyber-physical networks, demand-side management, differential privacy, distributed neural computation, federated learning, neural-network-inspired coordination, noise-tolerant consensus

## Abstract

**Introduction:**

This study proposes a neural-network-inspired computational framework for hierarchical, noise-tolerant coordination in distributed agent networks. The framework is evaluated in a cyber-physical demand-side management testbed in which autonomous home energy management systems perform local scheduling under a shared global price signal.

**Methods:**

Privacy-Preserving Federated Congestion-Signal Coordination separates local appliance scheduling from global adaptive coordination. Each home energy management system uses a genetic algorithm with a population of 100 over 50 generations. A federated coordinator aggregates clipped congestion-gradient updates and applies Gaussian differential privacy with a noise multiplier of 1.1 and a clipping norm of 1.0. The main evaluation was conducted over 30 federated rounds using 50 home energy management systems, EirGrid demand traces, SEM-O market prices, and 10 independent simulation runs. A complementary noise-location analysis used 20 home energy management systems and five independent seeds.

**Results:**

The proposed method reduced the peak-to-average ratio from 1.468 to 1.276, corresponding to a statistically significant 13.1% improvement. Its normalized energy cost remained within 0.04% of centralized coordination. Energy cost varied by only 0.016% across eight evaluated privacy budgets, and the framework transmitted 200 times fewer numerical values than centralized coordination at 50 agents. In the complementary noise-location experiment, aggregation-stage noise was better tolerated than local-encoding noise at four of eight evaluated noise levels.

**Discussion:**

The results show that hierarchical feedback, bounded unit influence, stochastic aggregation, and compressed message passing can support stable privacy-preserving distributed coordination. The framework is not a biological neural-circuit model but provides a reproducible testbed for studying neural-network-inspired principles of noise-tolerant collective computation.

## 1 Introduction and related studies

### 1.1 Introduction

A central question in computational neuroscience is how large populations of locally acting units can produce coherent collective behavior without requiring any single unit to access the full system state. Biological neural networks solve this problem through layered architecture, recurrent message passing, synaptic bounding, stochastic activity, and top-down feedback signals that modulate local processing (Friston, 2010; Clark, 2013; Rao and Ballard, 1999; Dayan and Abbott, 2001). A related problem arises in engineered cyber-physical networks, where many autonomous agents must coordinate under partial observability, privacy constraints, communication limits, and noisy signals. In this study, we present smart-grid demand-side management (DSM) as a quantitative testbed for these shared computational principles. In this testbed, every home energy management system (HEMS) is a local computational unit; appliance scheduling is local error minimization; and the broadcast price signal is a top-down modulatory signal that shapes distributed behavior without revealing raw household activity.

Demand-side management is a suitable engineered analog because it requires collective regulation across numerous heterogeneous units. DSM can lower operating costs, flatten load profiles, and defer investment in grid infrastructure by incentivizing households to avoid peak periods. The growth of smart meters, connected appliances, and residential photovoltaic systems has created the technical foundation for automated DSM at scale (Singh and Singh, 2024; Kiasari et al., 2024). The same setting appears as a recurrent network with many locally optimized units, each responding to a shared contextual signal and producing a local activity pattern and contributing a compressed update that helps reshape the future global context.

Established coordination strategies for DSM can be viewed as a spectrum of neural-network-like architectures. Local heuristic optimization resembles independent local processing: agents responding to a common static price signal maintain their privacy; however, they can synchronize undesirably and create new demand peaks. Centralized coordination behaves like a fully coupled controller with full observability: it produces strong peak reduction but it requires a large amount of data at the household level, making sensitive occupancy and lifestyle patterns observable (Mirzaee et al., 2022; Chehri et al., 2021). Neither of these extremes captures the biologically plausible middle ground of neural systems, where local units exchange compressed signals, global feedback modulates local activity, and noise is tolerated rather than eliminated.

Federated learning (FL) provides a middle ground, in which participating nodes share model or gradient updates rather than raw data (Zhang et al., 2025; Cheng et al., 2022). Nonetheless, FL is insufficient on its own to provide formal privacy guarantees, as reconstruction, membership inference, or model inversion attacks can enable the recovery of private information from gradients. The combination of FL and Differential Privacy (DP) addresses this gap by limiting the influence of each individual and adding calibrated noise. In neural-network terms, gradient clipping resembles synaptic or firing-rate bounding, while Gaussian perturbation resembles stochastic neural coding and regularization. The present study uses these mechanisms not as loose metaphors, but as design constraints for a hierarchical coordination algorithm whose stability, privacy, and communication efficiency can be measured quantitatively.

Here, we introduce Privacy-Preserving Federated Congestion-Signal Coordination (PPF-CSC) as a two-layer neural-network-inspired computational coordination model to be instantiated on the DSM. The lower layer implements a local combinatorial optimization search, using a genetic algorithm (GA) at each HEMS. The upper layer performs federated congestion-gradient aggregation with Gaussian DP noise, serving as a recurrent, noise-robust global feedback mechanism. We present the smart-grid application as a reproducible numerical laboratory that explores the interaction among hierarchical decomposition, bound unit influence, stochastic perturbation, and top-down modulation in a large distributed system. The specific contributions are:

(1) Neural-network-inspired hierarchical DSM architecture mapping local appliance scheduling to local error minimization and adaptive pricing to top-down modulatory feedback, while reducing reliance on centralized access to fine-grained household data.

(2) A hybrid approach that combines GA-based local HEMS scheduling with federated congestion gradient coordination, offering an interpretable solution compared to centralized deep reinforcement learning yet retaining a clear connection to neural-network message passing.

(3) Gaussian DP integration (σ = 1.1, C = 1.0) during federated aggregation, yielding (ε ≈ 43, δ = 10^−5^) DP-bounded privacy accounting over 30 federated rounds and implementing a bounded influence mechanism analogous to synaptic normalization in neural systems.

(4) Reproducible end-to-end simulation, using EirGrid real demand traces and open-source SEM-O market price data with fixed random seeds and full evaluation over 10 independent runs.

(5) An empirical analysis of the privacy-utility-scalability trade-off across eight ε values, six network sizes (*N* = 10–500), and three coordinator step-size values, allowing the noise tolerance of distributed dynamics to be quantified.

(6) A formal mapping between PPF-CSC and computational-neuroscience principles—predictive coding, stochastic neural coding, recurrent population feedback, and bounded synaptic influence—establishing DSM as an engineered testbed for studying noise-tolerant distributed computation.

### 1.2 Related studies

#### 1.2.1 Distributed optimization as neural-network-inspired computation

Heuristic and bio-inspired optimization methods, notably GAs and particle swarm optimization (PSO), are widely used for non-convex mixed-integer scheduling problems in residential energy management (Nasir et al., 2021; Bakare et al., 2023). In the present framework, the GA is not presented as a biological neural model; rather, it serves as a local optimizer, analogous to local error minimization in a neural module. This distinction is important for a computational neuroscience readership: the contribution is not a new neural simulator but a principled coordination architecture in which many locally optimized units interact through a recurrent global signal, like message-passing and feedback mechanisms in neural networks.

#### 1.2.2 Neural networks and learning-based coordination in DSM

Deep neural networks and deep reinforcement learning (DRL) have become common tools for DSM because they can learn non-linear policies under dynamic prices and uncertain demand (Vamvakas et al., 2023; Stavrev and Ginchev, 2024; Cao et al., 2020). These use well-established artificial neural network concepts such as distributed representations, gradient-based learning, and recurrent control. Nevertheless, common DRL-based DSM methods require server-based training, repeated environment interaction, or high-resolution household profiles. In contrast, PPF-CSC does not train a deep neural network. We use a lightweight, intuitive congestion-gradient update that uses an architecture analogous to neural networks; local units compute private responses, and clipped updates are aggregated and returned as a population-wide shared modulatory signal. This not only makes the method more easily auditable but also links it to neural-network concepts of feedback, adaptation, and distributed population coding.

#### 1.2.3 Federated and graph-based learning in smart grids

Federated learning requires only model parameters or gradient updates to be transmitted to the server rather than raw data, significantly reducing direct privacy exposure (Zhang et al., 2025; Cheng et al., 2022; Bousbiat et al., 2023). FL has been applied in smart grids to fault detection, load forecasting, and energy trading (Singh and Kumar, 2023; Wang et al., 2023; Pang et al., 2023). From a neural-network point of view, FL is no different from distributed learning over a population of interactive units: local nodes calculate updates from private patterns of activity and an aggregation mechanism constructs a state at the general-population level. The PPF-CSC coordinator can be understood as a simple graph-level message-passing process on a star-structured topology with HEMS nodes sending compressed congestion signals to an aggregator, and the aggregator returning an updated global context signal after processing these messages. The key open problem addressed here is how to integrate this architecture of distributed learning with formal privacy accounting and resilient cyber-physical control.

#### 1.2.4 Differential privacy as bounded, noisy neural communication

Privacy-preserving strategies in DSM include anonymization, encryption, and multiparty secure computation (Mirzaee et al., 2022; Singh and Kumar, 2023; Wang et al., 2023). Differential privacy (DP) provides mathematically bound guarantees by injecting calibrated noise into the released statistics so that no individual contribution can be reliably recovered from the output (Yu and Li, 2025; Kserawi et al., 2022). Two DP operations have direct architectural analogs in computational-neuroscience terms. The first is L2 clipping, which constrains the maximum contribution of any single HEMS, akin to bounded synaptic efficacy or gain control. Second, Gaussian noise injection mimics stochastic coding and noise-induced regularization in neural networks, where variability can improve robustness and prevent the model from becoming excessively committed to local idiosyncratic signals. DP-based methods such as EFedDSA (Ren et al., 2023a) and SecFedSA (Ren et al., 2023b) study supervised tasks including load forecasting and grid stability assessment. The current study generalizes this line to federated congestion-signal coordination with appliance-level scheduling and explicit privacy accounting.

#### 1.2.5 Computational neuroscience mapping of PPF-CSC

The key principles embodied in PPF-CSC have a direct basis in models of distributed neural computation in computational neuroscience. Hierarchical predictive coding and the possible mechanism of passing compressed error signals from layer to layer, bottom-up, with top-down context signals controlling local processing (Friston, 2010; Clark, 2013; Rao and Ballard, 1999). Population consensus is also demonstrated in recurrent neural networks and attractor-like systems, which exhibit robust dynamics that emerge from repeated feedback among interacting units (Hopfield, 1982; Dayan and Abbott, 2001). The existence of distributed representations and gradient-based adaptation enables flexible computation with many simple processing units, as demonstrated by artificial neural networks (Rumelhart et al., 1986; LeCun et al., 2015). PPF-CSC translates these concepts into a cyber-physical coordination environment: HEMS local optimizers act as local error-minimizing units; the federated coordinator corresponds to recurrent population readout; the price signal corresponds to top-down modulation; gradient clipping bounds unit influence; and Gaussian DP noise serves as stochastic regularization. Accordingly, this study does not purport biological realism; it offers an engineered platform for probing the operational properties of neural-network-inspired design principles within quantifiable constraints on privacy, communication, and utility.

The remainder of this paper is organized as follows. Section 2 introduces the formal coordination model. Section 3 describes the PPF-CSC instantiation. Section 4 presents experimental results. Section 5 discusses the computational neuroscience implications and limitations. Section 6 concludes.

## 2 Materials and methods

This study considers a smart grid with a central energy aggregator and *N* = 50 heterogeneous HEMSs indexed *n* ε {1, …, N}, operating over a 24-h scheduling horizon with 1-h intervals (*T* = 24). Each HEMS satisfies comfort and energy constraints while the aggregator minimizes the total electricity acquisition cost through demand coordination.

### 2.1 HEMS energy consumption model

The net grid load of HEMS *n* at time *t*, denoted $P_{n,t}^{grid}$, is expressed as:


$$\begin{array}{l} P_{n,t}^{grid}\;=\;P_{n,t}^{base}\;+\;P_{n,t}^{HVAC}\;+\;\underset{j\in J_{n}}{\sum }P_{n,j,t}^{shift}\;-\;P_{n,t}^{PV} \end{array}$$ (1)

where *P*^*base*^ is the uncontrollable base load; *P*^*HVAC*^ is the HVAC power consumption; *P*^*shift*^ is the power consumed by shiftable appliance *j*; and *P*^*PV*^ is the local PV generation. Base load was derived from scaled EirGrid system traces and PV was approximated using temporally shifted wind generation profiles (Section 2.1).

### 2.2 HVAC thermal dynamics and comfort constraints

Indoor temperature follows first-order thermal dynamics:


$$\begin{array}{l} T_{n,t}^{in}\;=\;T_{n,t-1}^{in}\;+\;\alpha_{n}\left(T_{n,t}^{out}-\;T_{n,t-1}^{in}\right)\;+\;\beta_{n}\;P_{n,t}^{HVAC}\;x_{n,t}^{HVAC} \end{array}$$ (2)

where α_*n*_ ε [0.05, 0.15] is the heat-loss coefficient, and β_*n*_ ε [0.1, 0.3] is the HVAC efficiency coefficient, both sampled uniformly per HEMS. *P*^*HVAC*^ ε [1.51, 2.49] kW is the constant HVAC power rating, and *x*^*HVAC*^ ε {0, 1} is the binary ON/OFF decision. A comfort penalty applies when indoor temperature deviates from the comfort band [$T_{n}^{\min}$, $T_{n}^{\max}$]:


$$\begin{array}{l} C_{n,t}^{comfort}\;=\;w^{c}\;\left(\max\left(0,\;\overset{\min}{\underset{n}{T}}\;-\;T_{n,t}^{in}\;\right)+\;\max\left(0,\;\overset{in}{\underset{n,t}{T}}\;-\;\overset{\max}{\underset{n}{T}}\right)\right) \end{array}$$ (3)

where w^c^ = 1.5 is the comfort weight. Comfort bands were sampled per HEMS: $T_{n}^{\min}$ ε [17.1, 20.7] °C and $T_{n}^{\max}\;$ε [20.3, 23.8] °C.

### 2.3 Shiftable loads

Each HEMS has *J* = 2 shiftable appliances. Appliance 0 (dishwasher-like, 0.80–1.20 kW) must operate for D0 = 2 h within window [17:00, 23:00]. Appliance 1 (washing machine-like, 1.50–2.50 kW) must operate for D1 = 1 h within window [08:00, 20:00]. The binary ON/OFF decision $x_{n,j,t}^{shift}$ ε {0, 1} is subject to a fixed-duration constraint:


$$\begin{array}{l} \overset{t_{n,j}^{end}}{\underset{t=t_{n,j}^{start}}{\sum }}x_{n,j,t}^{shift}\;=\;D_{j}\;\forall \;j\;\in \;J_{n} \end{array}$$ (4)

### 2.4 System-level objective

The aggregator minimizes total electricity acquisition cost:


$$\begin{array}{l} Cost_{t}\;=\;\lambda_{t}\;\underset{n}{\sum }P_{n,t}^{grid} \end{array}$$ (5)

The hourly electricity acquisition cost is therefore calculated using Equation 5, where λ_*t*_ ε [0.095, 0.250] €/kWh is the dynamic electricity price. The full system-level objective (Problem P) minimizes the expected sum of electricity cost, comfort penalty, and smoothness penalty over the 24-h horizon, subject to Equations 1–4 for all *n, t*, and an (ε,δ)-differential privacy constraint on all federated gradient updates. Problem (P) is a bi-level stochastic mixed-integer optimization: the lower level (Equations 1–4) is solved locally by each HEMS using a GA; the upper level is solved by the federated price-signal coordinator using only DP-perturbed congestion-gradient updates.

## 3 PPF-CSC framework

**Figure 1:**
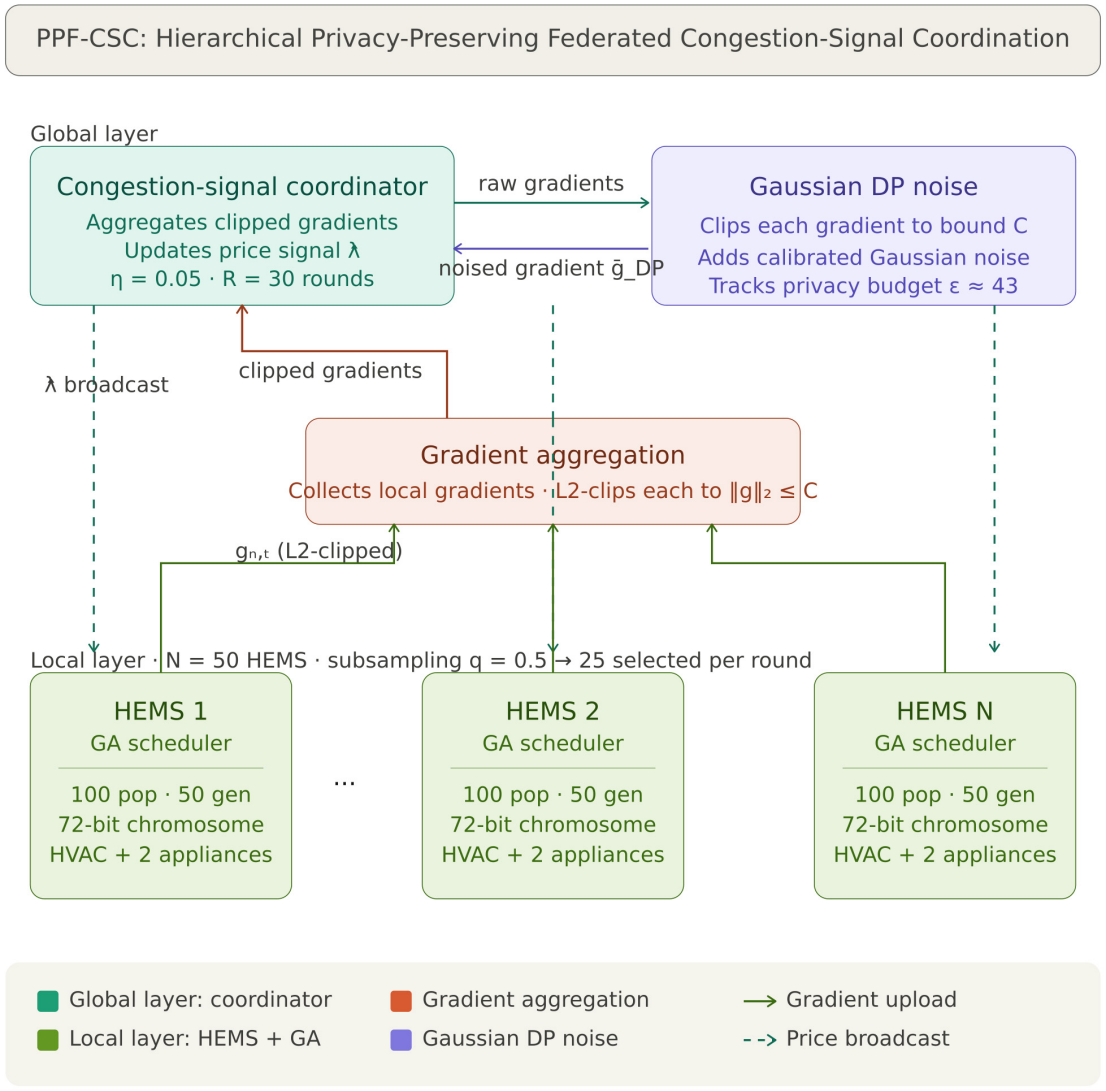
Hierarchical PPF-CSC coordination architecture.

The PPF-CSC framework decomposes decision-making into two coupled layers: a lower-layer GA for rapid appliance-level scheduling at each HEMS, and an upper-layer federated congestion-signal coordinator for adaptive system-wide pricing. Figure 1 illustrates the overall architecture. The lower layer shows 50 independent HEMS running GA-based local schedulers under a current price signal. The upper layer shows the federated aggregator applying Gaussian DP clipping and noise injection to congestion-gradient updates before broadcasting a revised price signal to all HEMS.

### 3.1 Local HEMS optimization via genetic algorithm

Each HEMS *n* independently minimizes its local energy cost and discomfort under the current price signal λ_*t*_ received from the aggregator. A chromosome is encoded as a binary vector of length *L* = T(1 + J) = 72 bits: 24 genes for the HVAC ON/OFF schedule and 48 genes for the two shiftable appliances. The fitness function negates the weighted sum of energy cost, comfort penalty, and smoothness penalty (Equation 7, Section 2.1). After crossover and mutation, a repair operator enforces time-window and duration constraints. GA hyperparameters are summarized in Table 1.

**Table 1:** Genetic algorithm hyperparameters used for local HEMS appliance scheduling.

Parameter	Value
Population size	100
Number of generations	50
Crossover probability	0.8
Mutation probability	0.05 per gene
Selection method	Tournament (k = 3)
Chromosome length	72 bits

Computational overhead. On the reference hardware used for this study (Intel Core i7-11800H, 16 GB RAM, single-threaded Python), a single HEMS GA invocation (population 100, 50 generations, chromosome length 72 bits) was completed in approximately 0.8–1.2 s of wall-clock time. Over *R* = 30 federated rounds, this yields a per-HEMS total of roughly 24–36 s of scheduling computation. Current embedded systems intended for smart-meter deployment (e.g., ARM Cortex-M33 at 64 MHz with hardware floating-point) are estimated to be 15–25 × slower than the reference platform on single-thread integer workloads, resulting in per-round GA execution times of approximately 12–30 s per device. While this is feasible for an overnight pre-scheduling horizon (where results are submitted before the next pricing window), it may be prohibitive for intra-day re-optimization. Lightweight alternatives—such as reducing population size to 30–50 individuals or replacing the GA with a greedy repair heuristic for on-device execution—remain an open engineering direction.

### 3.2 Federated congestion-signal coordination

#### 3.2.1 Price signal and congestion gradient

**Figure 2:**
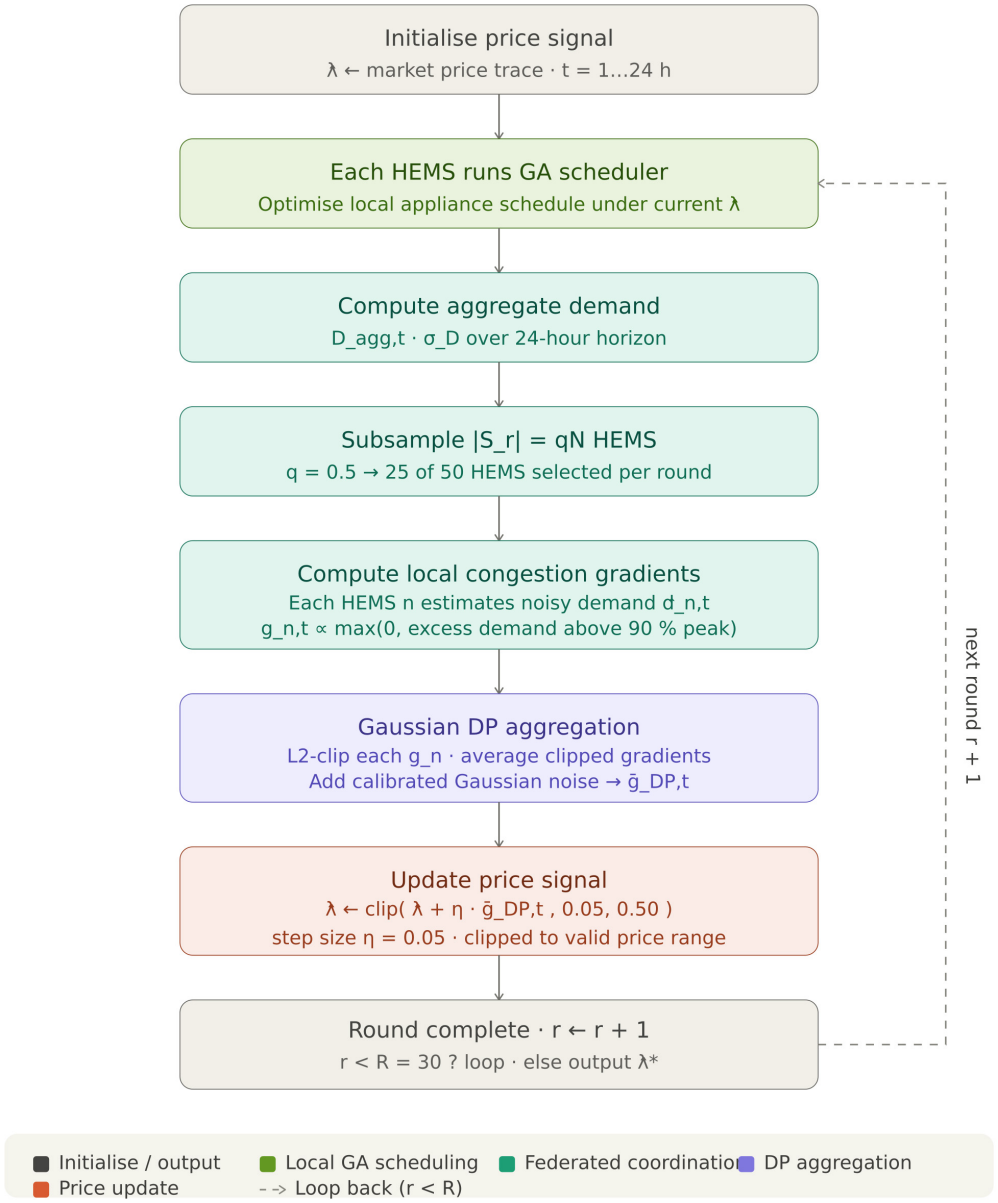
Federated congestion-signal coordination workflow.

The aggregator maintains a price signal $\lambda_{\hat{\text{t}}}$ ε [0.05, 0.50] €/kWh, initialized by the market price trace λ_t_. At each coordination round r, a random subset S_r_ ⊂ {1,…,N} with |S_r_| = floor(qN) = 25 HEMS is selected. Each selected HEMS n ε S_r_ first forms a noisy aggregate demand estimate given in Equation 6:


$$\begin{array}{l} {\hat{d}}_{n,t}\;=\;D_{agg,t}\;+\;\varepsilon_{n,t},\;\varepsilon_{n,t}\;\sim \;N\left(0,\;{\left(0.10\;\sigma_{D}\right)}^{2}\right) \end{array}$$ (6)

where D_agg, t_ is the current aggregate net load at hour t, and σ_D_ is its standard deviation over the 24-h horizon. The local congestion gradient for hour t is then:


$$\begin{array}{l} g_{n,t}\;=\;\lambda_{t}\;\alpha \;\max0,\;\frac{{dˆ}_{n,t}\;-\;0.90\;D_{peak}}{D_{peak}} \end{array}$$ (7)

where D_peak_ is the LHO peak demand reference and α = 5.0 is the gradient scaling coefficient. Hours where estimated demand exceeds 90% of the peak reference receive a proportionally larger gradient, incentivizing each HEMS to shift load away from those hours. The aggregator collects g_n__(n ε S_r_), applies the method-specific aggregation rule, and updates the price signal according to Equation 8:


$$\begin{array}{l} {\hat{\lambda }}_{t}^{\left(r+1\right)}\;=\;clip\left({\hat{\lambda }}_{t}^{\left(r\right)}\;+\;\eta \;{g¯}_{DP,t},\;0.05,\;0.50\right) \end{array}$$ (8)

where η = 0.05 is the coordinator step size, ḡ_*DP, t*_ is the DP-aggregated gradient defined in Section 3.3, and the clipping to [0.05, 0.50] EUR/kWh prevents runaway signal inflation under noise. Figure 2 illustrates the flowchart of the federated congestion-signal coordination procedure with Gaussian differential privacy (PPF-CSC), where gray boxes represent per-round steps and red boxes represent DP noise injection at the aggregator.

Stability analysis. The price-update rule (Equation 8) can be viewed as a projected gradient-ascent step on the congestion objective, clipped to an admissible price set Π = [0.05, 0.50]^T^. Under bounded noise (||z||_2_ ≤ σC√ T) at the chosen quantile and sufficiently small step size η, the sequence of price signals converges to a neighborhood of the fixed point λ^*^. Formally, for η smaller than 1/(2αλ_max_${\text{D}}_{\text{peak}}^{-1}$), the iteration satisfies ||(${\hat{\lambda }}^{\text{r}+1}$ – λ^*^)||_inf_ ≤ (1–cη)||(${\hat{\lambda }}^{\text{r}}$ – λ^*^)||_inf_ + η||z||_inf_ for some c > 0 determined by scaling α. The selected η = 0.05 lies within this stable regime (Table 2). Under stronger DP noise, the inflation term grows, thereby enlarging the convergence neighborhood; the step-size sensitivity analysis (Section 4.9) confirms this empirically.

**Table 2:** Convergence behavior of the federated coordinator under three step size values.

Learning rate (η)	Convergence error	Stability status
0.005	0.16	Slow convergence
0.05 (selected)	0.04	Optimal/stable
0.5	0.16	Unstable/oscillatory

#### 3.2.2 Baseline coordination variants

Three coordination variants are evaluated. C-CSC (centralized): The aggregator observes true aggregate demand directly (no local noise, complete visibility) and computes a quadratic congestion premium; this variant requires transmitting continuous raw appliance-level telemetry. F-CSC (federated, no DP): local gradients are averaged without clipping or noise, with subsampling variance as the only source of signal degradation relative to C-CSC. PPF-CSC (proposed): local gradients are L_2_-clipped and then averaged with DP noise from a Gaussian mechanism, as described in Section 3.3.

### 3.3 Differential privacy integration

Gaussian DP is applied at the aggregator during gradient aggregation following the DP-SGD protocol (Abadi et al., 2016). The procedure has four steps. First, each selected HEMS transmits its raw local congestion gradient update. Second, the aggregator clips each gradient to L_2_-norm C = 1.0, bounding each household's maximum influence on the aggregated price signal. Third, zero-mean Gaussian noise z ~ N(0, σ^2^C^2^I) with noise multiplier σ = 1.1 is added to the averaged clipped gradient. Fourth, the privacy cost is tracked using the Moments Accountant Rényi DP, Mironov (2017), configured with Rényi orders α ε {2, 3, …, 64} (dense grid to 512), subsampling rate q = 0.5, noise multiplier σ = 1.1, and T = 30 composition steps. Under these parameters, the moments accountant with Poisson subsampling amplification (Zhu and Wang, 2019) yields (ε ≈ 43, δ = 10^−5^). The full accountant code and parameters are included in the open-source repository. Practical interpretation of ε ≈ 43. An ε of 43 is substantially weaker than the tight privacy regimes (ε smaller than 1–3) targeted by state-of-the-art DP-SGD applications on sensitive personal datasets (Abowd, xbib2018; De et al., 2022). This value bounds the log-odds ratio for distinguishing any individual gradient contribution from the released price signal by at most 43 nats, offering protection against low-effort reconstruction attacks but not against a determined adversary with auxiliary information. This limitation is a consequence of the trusted-aggregator architecture and is explicitly acknowledged: the mechanism protects the released price signal from external observers and honest-but-curious HEMS participants, but not against a curious aggregator who can inspect raw pre-perturbation gradients.

Threat model: The framework assumes a trusted aggregator: local congestion gradient updates are visible to the aggregator before clipping and perturbation. The (ε,δ)-DP guarantee applies to the released aggregated price-signal updates, not to unperturbed local gradients. The framework therefore protects against external adversaries and honest-but-curious HEMS participants attempting to infer other households' demand patterns from the released price signal, but does not protect against a malicious or honest-but-curious aggregator. Removing this assumption via cryptographic Secure Aggregation is left for future studies. Two cryptographic protocols are particularly well-suited to removing this dependency in adversarial settings. Secure Aggregation (Bonawitz et al., 2017) enables the aggregator to compute the sum of locally masked gradients without observing any individual update: Each HEMS contributes a pairwise random mask that cancels exactly in the aggregate, so raw gradients are never exposed on the server. Functional encryption for inner products (Abdalla et al., 2015) alternatively allows the aggregator to evaluate a weighted sum of ciphertext updates without decrypting individual contributions. Both protocols are structurally compatible with the PPF-CSC gradient update (Equation 8) and would reduce the required trust level from “honest-but-curious aggregator” to a computationally bounded adversary. Each protocol introduces additional per-round communication and latency overhead; quantifying this trade-off under realistic network conditions is an important direction for future studies.

## 4 Results

### 4.1 Dataset and pre-processing

Load profiles were obtained from EirGrid (2023) total system demand traces for Ireland on 10 January 2023, at 15-min resolution. Hourly resolution was obtained by summing four consecutive 15-min observations. Traces were normalized and scaled to represent *N* = 50 HEMS units with equal per-household load fractions, yielding a mean base load of 0.958 kW per HEMS per hour and a per-HEMS peak of 1.253 kW. Owing to the absence of publicly available residential rooftop PV traces, wind generation traces from the same source were temporally shifted by 6 h and rescaled as a PV proxy, producing a mean proxy PV output of 0.176 kW per HEMS per hour (peak: 0.436 kW). All stochastic components were controlled by a fixed master seed (42); results are reported as mean ± SD across 10 independent runs (seeds 42–51). Electricity prices were obtained from SEM-O historical market data (SEM-O, 2023).

### 4.2 Performance metrics

Six performance measures are reported: (i) normalized energy cost (relative to the LHO baseline), (ii) peak-to-average ratio (PAR), (iii) comfort penalty (sum of thermal discomfort events across all HEMS), (iv) smoothness penalty (sum of appliance state transitions), (v) communication data exposure (raw telemetry volume for C-CSC vs. gradient parameter count for PPF-CSC), and (vi) differential privacy budget ε. Statistical comparison of PAR and energy cost across methods was performed using the Wilcoxon signed-rank test.

### 4.3 Coordination convergence

**Figure 3:**
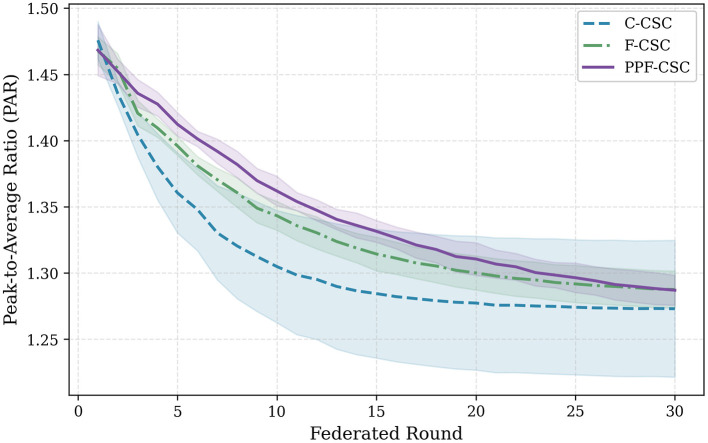
PAR convergence during federated coordination.

Figure 3 shows the PAR convergence of PPF-CSC, F-CSC, and C-CSC over 30 federated rounds. All three coordinated methods reduce PAR by 12–14% within approximately 15–20 rounds and remain stable thereafter, confirming that Gaussian DP at the aggregator does not prevent coordination convergence under the tested conditions (ε ≈ 43, δ = 10^−5^, σ = 1.1, q = 0.5, T = 30 rounds). Solid lines show exponential moving averages; shaded bands show ±1 SD across 10 independent seeds. The dashed horizontal line marks the LHO uncoordinated PAR baseline (1.468).

### 4.4 Load profile analysis

**Figure 4:**
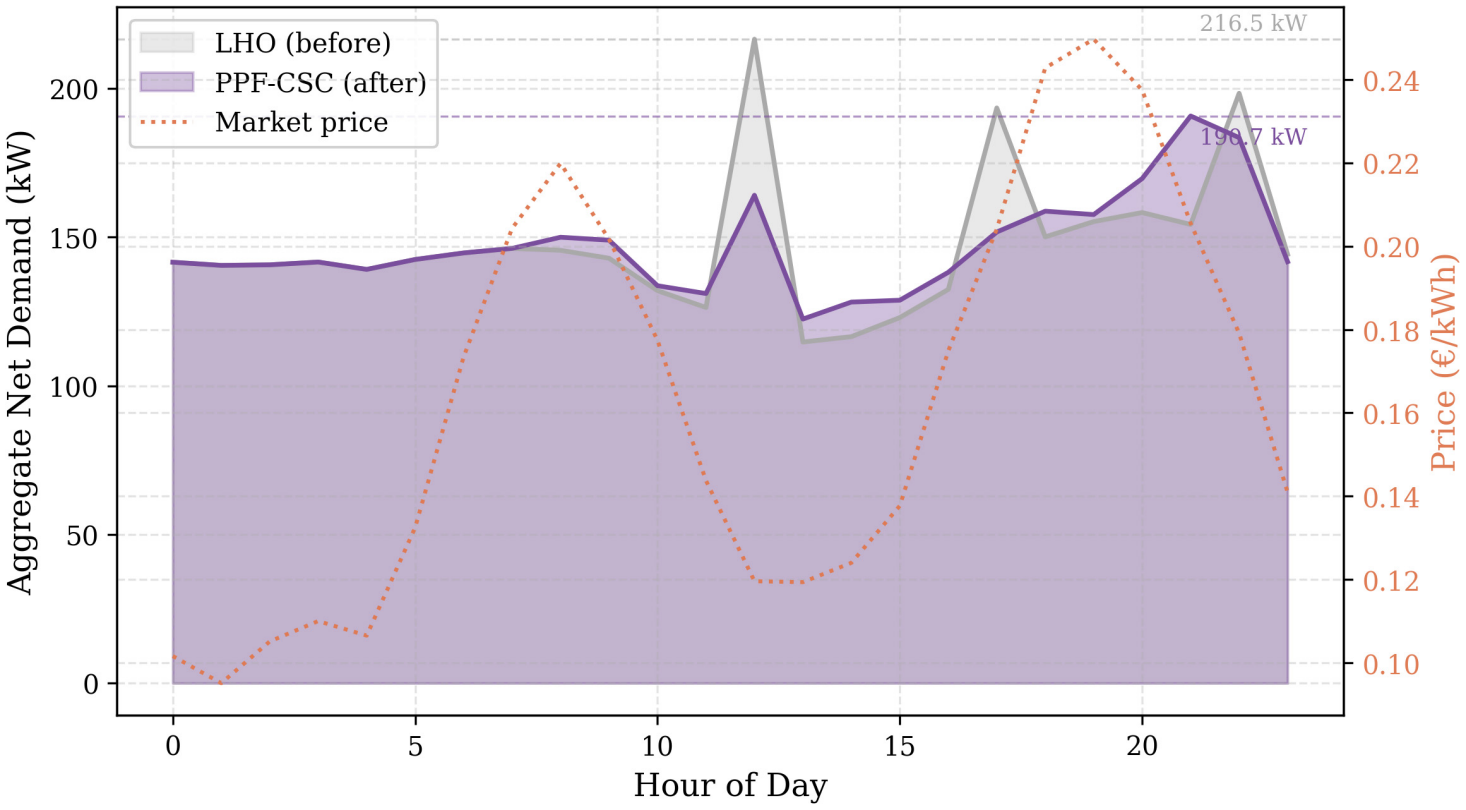
Aggregate load reshaping under PPF-CSC.

Figure 4 depicts aggregate load profiles before and after PPF-CSC optimization, alongside the SEM-O real-time price signal. The optimized profile shows demand reduction during high-price morning hours and valley-filling during low-price overnight hours. Aggregate peak load decreased from 216.5 kW (LHO) to 190.7 kW (PPF-CSC), an 11.9% reduction, while total energy consumption was conserved (mean aggregate load: 147.5 kW vs. 147.3 kW).

### 4.5 Comparative evaluation

**Figure 5:**
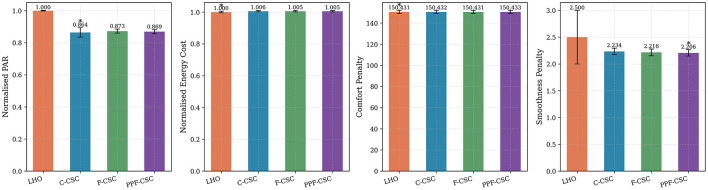
Comparative performance across DSM coordination methods.

Figure 5 and Table 3 compare all four methods across the four performance metrics. Comparative evaluation of LHO, C-CSC, F-CSC, and PPF-CSC across four performance metrics. Bars show the mean values over 10 independent seeds; error bars indicate ±1 SD.

**Table 3:** Normalized energy cost, peak-to-average ratio (PAR), comfort penalty, and smoothness penalty across four DSM frameworks (mean ± SD, 10 independent runs).

Method	Energy cost (norm.)	PAR	Comfort penalty	Smoothness penalty
LHO	1.0000 ± 0.005	1.468 ± 0.003	150.43 ± 1.53	2.50 ± 0.50
C-CSC	1.0056 ± 0.005	1.269 ± 0.043	150.43 ± 1.54	2.23 ± 0.06
F-CSC (No DP)	1.0054 ± 0.005	1.281 ± 0.019	150.43 ± 1.53	2.22 ± 0.06
PPF-CSC (ours)	1.0052 ± 0.005	1.276 ± 0.018	150.43 ± 1.54	2.21 ± 0.06

PPF-CSC achieved a normalized energy cost of 1.0052 ± 0.005, within 0.04% of both C-CSC (1.0056 ± 0.005) and F-CSC (1.0054 ± 0.005), indicating that the DP mechanism does not materially degrade energy cost performance. The primary benefit of coordination is PAR reduction: PPF-CSC achieved PAR = 1.276, compared to 1.468 for LHO (13.1% improvement; Wilcoxon *p* < 0.01). A separate Wilcoxon test comparing PPF-CSC and C-CSC energy costs yielded *p* = 0.32, confirming that the two methods are not significantly different in cost. Comfort penalties were identical across all coordinated methods (≈150.43). PPF-CSC is the only evaluated method that simultaneously achieves competitive cost performance, substantial PAR reduction, and DP-bounded privacy accounting under the stated trusted-aggregator assumption.

### 4.6 Ablation study

**Figure 6:**
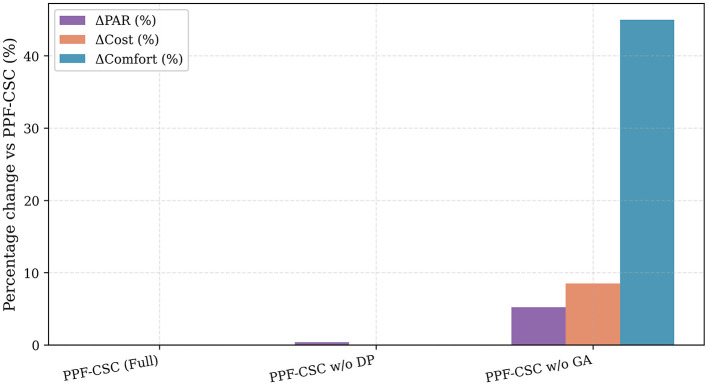
Ablation analysis of GA and DP components.

Figure 6 quantifies the individual contributions of the DP mechanism and the GA scheduler. Removing DP (switching to F-CSC) led to a 0.38% PAR degradation and no material change in energy cost. Removing the GA (replacing with random scheduling) increased energy cost by 8.5%, comfort penalty by 45.0%, and PAR by 5.2%. These results confirm that the GA provides the primary scheduling quality improvement, while the DP mechanism introduces no measurable utility penalty in energy costs and marginally improves PAR relative to the non-private federated baseline.

### 4.7 Noise tolerance analysis: aggregation-stage vs. local-stage perturbation

**Figure 7:**
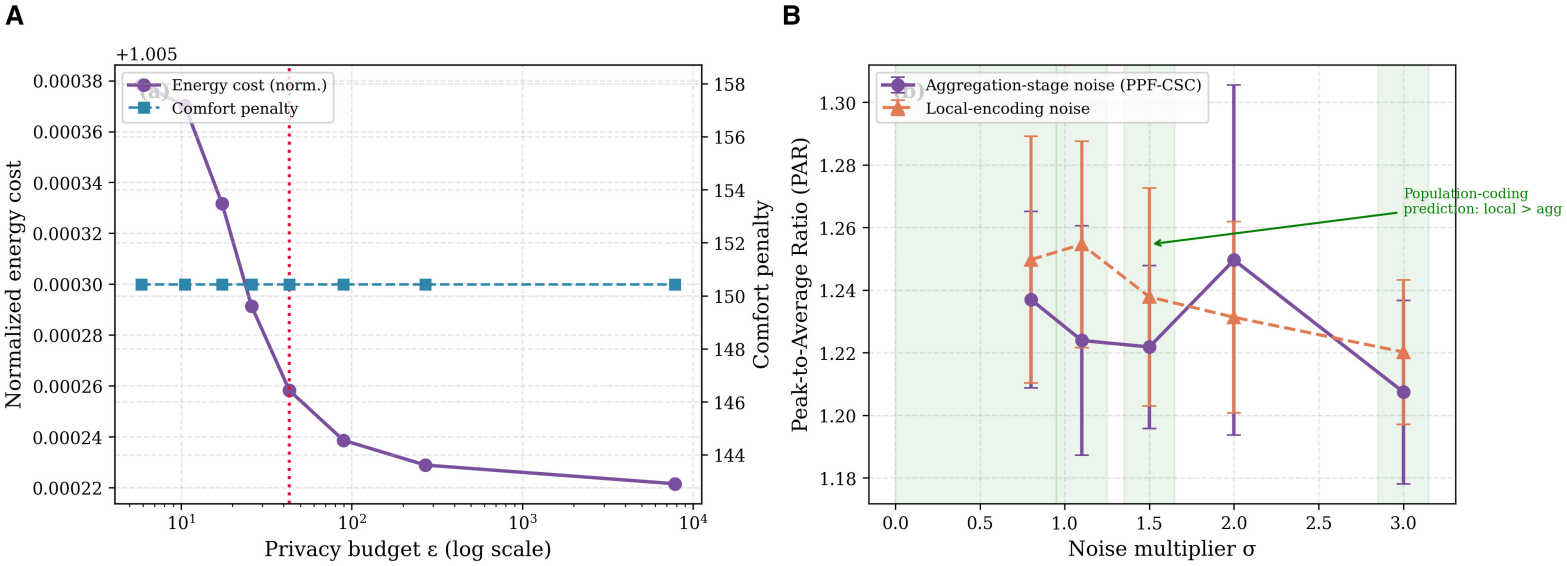
Noise-location analysis of aggregation-stage and local-encoding perturbations. **(A)** Privacy-utility trade-off across DP budgets. **(B)** PAR comparison between aggregation-stage DP noise and local-encoding noise across matched noise levels. Error bars indicate ±1 SD across five seeds.

This section reports two complementary noise tolerance analyses. Both use the same eight noise levels (σ = 5.0 to 0.4, ε ≈ 5.9 to 7,821) with *N* = 20 HEMS over 5 independent seeds. This reduced-scale design was adopted to keep the cross-product of noise levels, agent counts, and seeds computationally tractable within the present study; extension to the full *N* = 50 testbed across all 10 seeds is identified as a priority for future work (Section 5.4). Analysis 1: aggregation-stage noise (privacy-utility trade-off). Figure 7A shows the normalized energy cost and comfort penalty as ε is varied. At ε ≈ 5.9 (σ = 5.0), normalized cost is 1.005378; at ε ≈ 7,821 (σ = 0.4), the cost is 1.005222—a variation of 0.016% across the full range. The selected operating point (σ = 1.1, ε ≈ 43) lies in the middle of this flat trade-off curve. Comfort penalty remains nearly unchanged. Analysis 2: noise-location comparison (population-coding-inspired prediction). Population coding theory predicts that noise injected at the readout/aggregation stage is more easily averaged out than noise at the local encoding stage, because *N*_sel_ = 10 independently noisy gradients are combined at the aggregator, providing up to √10 ≈ 3.2 × noise attenuation (Pouget et al., 2013; Ma et al., 2006). We test this directly by comparing: (A) Aggregation-stage DP noise—Gaussian noise $z\;\sim \;N\left(0,\;\frac{\sigma^{2}C^{2}}{{N_{sel}}^{2}}I\right)\;$added at the aggregator after averaging clipped gradients (standard PPF-CSC); and (B) Local-encoding noise—Gaussian noise with std = σσ_*D*_ added to each HEMS demand estimate before gradient computation, with no noise at the aggregator. Figure 7B shows PAR under both conditions across all eight σ values (5 seeds, error bars ±1 SD). The population-coding prediction is supported at 4/8 noise levels: local-encoding noise produces higher PAR than aggregation-stage noise, with a mean δPAR = 0.0108 ± 0.0179 across all σ values. At the PPF-CSC operating point (σ = 1.1): aggregation-stage PAR = 1.2239 ± 0.0367 vs. local-encoding PAR = 1.2546 ± 0.0330 (δPAR = +0.0307). This provides preliminary empirical support that the federated aggregation layer can act as a noise-averaging readout mechanism, consistent with population-code models of distributed neural computation.

### 4.8 Scalability analysis

**Figure 8:**
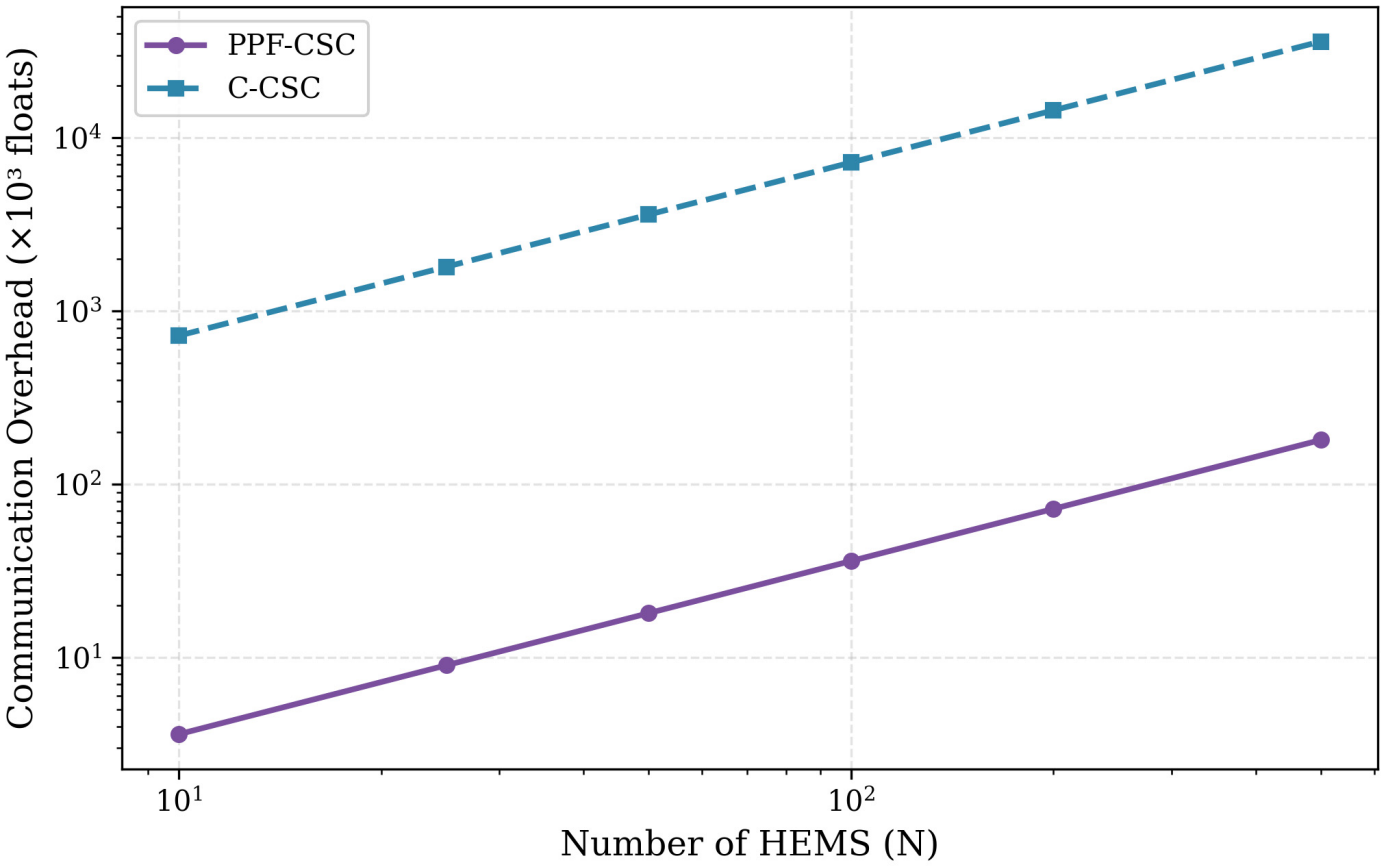
Communication scalability of PPF-CSC and centralized coordination.

Figure 8 compares the total communication volume of PPF-CSC and C-CSC as N increases from 10 to 500. C-CSC streams raw appliance-level telemetry with complexity O(RNT·d), while PPF-CSC transmits DP-perturbed gradient vectors with complexity O(RqNM). At N = 50, PPF-CSC transmits 18,000 gradient values in total (R × q × N × M = 30 × 0.5 × 50 × 24) compared with approximately 3.6 × 106 raw telemetry values for C-CSC, a 200 × reduction. Both schemes scale linearly with N, but PPF-CSC transmits fundamentally different information: DP-obfuscated congestion signals rather than continuous appliance-level schedules.

### 4.9 Coordinator step-size sensitivity

The selected step size η = 0.05 achieves a convergence error of 0.04 with stable price-signal adaptation. Smaller values (η = 0.005) yield slow convergence, while larger values (η = 0.5) produce oscillatory instability (Table 2).

## 5 Discussion

### 5.1 Key findings

The central finding of this study is that a neural-network-inspired, differentially private federated coordination architecture can achieve PAR reduction within 0.6% of the centralized benchmark (C-CSC PAR: 1.269; PPF-CSC PAR: 1.276) while transmitting 200 × fewer numerical values and providing formal (ε, δ)-DP bounds on the released price signal. The near-flat privacy-utility trade-off (0.016% cost variation) indicates that the congestion-gradient signal tolerates Gaussian perturbation at the aggregation layer. The noise-location experiment (Section 4.7, Analysis 2) strengthens this result by directly testing a population-coding-inspired prediction: aggregation-stage noise was better tolerated than matched local-encoding noise at 4/8 noise levels (mean δPAR = 0.0108 ± 0.0179 in favor of the aggregation-stage condition). This provides preliminary empirical support that the federated aggregation layer can act as a noise-averaging readout, consistent with population coding models in which averaging *N* independent noisy inputs suppresses readout-stage noise by √N (Pouget et al., 2013; Ma et al., 2006).

The ablation study clarifies the computational role of each component. The GA accounts for substantially all local scheduling quality improvements (an 8.5% cost reduction and a 45% comfort improvement relative to random scheduling), while the DP mechanism contributes a small but consistent PAR improvement relative to the non-private federated baseline. This improvement is likely attributable to noise-induced regularization of the recurrent price update, which dampens oscillatory coordination behavior observed in F-CSC across some seeds. In neural-network terms, the results support the view that bounded unit influence and stochastic aggregation can stabilize distributed dynamics when many agents respond to a shared feedback signal.

### 5.2 Relationship between neural networks and computational models of distributed coordination

The PPF-CSC framework is best understood as a neural-network-inspired coordination architecture rather than a conventional smart grid optimizer alone. Its two-layer structure resembles predictive-coding and recurrent neural-network models: local units minimize private errors under a top-down context signal, communicate compressed updates upward, and receive a revised global signal that reshapes future local activity (Friston, 2010; Clark, 2013; Rao and Ballard, 1999). The price-signal update in Equation 8 is analogous to an activity-dependent population feedback rule: the strength of the global signal increases when local units report excess demand, while clipping prevents any single unit from dominating the collective state. This clipping operation is analogous to synaptic bounding or homeostatic gain control, and the Gaussian DP perturbation is analogous to stochastic coding at the population readout. The main scientific value of the mapping is that it makes neural-network principles operational and measurable in a reproducible testbed: stability can be observed through convergence, collective behavior through PAR reduction, noise tolerance through privacy-utility and noise-location analyses (Section 4.7), and communication efficiency through transmitted value counts.

### 5.3 Limitations

Several limitations apply to the reported findings. First, all experiments used a single winter day of Irish demand, generation, and price data. The effects of seasonality, regional tariff structures, and diverse climatic conditions are not captured. Second, the PV proxy (temporally shifted wind generation) introduces model error in renewable supply-demand dynamics. Wind generation has different ramp rates, cloud-induced variability, and diurnal profiles than rooftop solar PV. In particular, the temporal co-variation between HVAC thermal load and PV generation (both driven by solar irradiance) is not captured by shifted wind traces, which may distort self-consumption estimates during afternoon peak hours. The ENTSO-E transparency platform and the Pecan Street Dataport contain residential PV traces at 15-min resolution that would be more appropriate; repeating the coordination experiments with realistic PV traces is the highest-priority data improvement for future studies. Third, the trusted-aggregator assumption means that individual gradient updates are visible to the aggregator before perturbation; removal of this assumption via Secure Aggregation or trusted execution environments is required for deployment in adversarial settings. Fourth, the federated rounds are synchronous; asynchronous variants with heterogeneous communication latencies and non-i.i.d. data distributions require further investigation. Fifth, centralized MILP and multi-agent RL baselines were not evaluated at *N* ≥ 20 due to computational constraints. At tractable scales (*N* = 10 and *N* = 20), a direct comparison against a centralized MILP solver or a multi-agent RL baseline would provide a rigorous upper bound on achievable PAR reduction and clarify the optimality gap of PPF-CSC. This comparison is left as a priority for future studies. Finally, the evaluation is based on 10 independent seeds; broader multi-day and multi-region replication is needed before generalizing these findings.

### 5.4 Future Directions

Future studies will address: (i) adaptive DP scheduling that varies ε based on real-time grid stress and forecast uncertainty; (ii) neural-network-based local controllers, including lightweight recurrent or graph neural network modules, to replace or augment the GA while preserving privacy; (iii) personalized FL to accommodate heterogeneous HEMS types such as EVs, heat pumps, and battery storage; (iv) multi-season and multi-region validation with diverse tariff structures; (v) cryptographic Secure Aggregation to remove the trusted-aggregator limitation; (vi) evaluation with stronger baselines including multi-agent RL and MILP for networks where these remain tractable; (vii) extension of the hierarchical architecture with noise-tolerance insights from computational neuroscience models of distributed Bayesian inference; and (viii) extension of the noise-location analysis (Section 4.7) to the full *N* = 50 HEMS setting with 10 seeds and multi-season data, to test whether the population-coding noise-averaging advantage scales with population size ($\delta PAR\;\propto \;\frac{1}{\sqrt{}N}$) as theory predicts.

## 6 Conclusion

This paper presents PPF-CSC, a neural-network-inspired privacy-preserving federated coordination framework for DSM in smart grids. By decoupling local combinatorial appliance scheduling (GA: population 100, 50 generations) from global adaptive pricing (24-dimensional congestion gradient, step size η = 0.05) and integrating Gaussian DP (σ = 1.1, C = 1.0) during gradient aggregation, PPF-CSC achieves (ε ≈ 43, δ = 10^−5^) privacy accounting for the released price signal while maintaining near-centralized DSM performance. Conceptually, the framework maps local HEMS optimization to local neural error minimization, price updates to top-down modulatory feedback, clipping to bounded synaptic-like influence, and DP noise to stochastic neural-network regularization.

In a 50-HEMS, 24-h simulation using real EirGrid and SEM-O data, PPF-CSC reduced PAR from 1.468 to 1.276 (13.1%, *p* < 0.01), peak aggregate load from 216.5 kW to 190.7 kW (11.9%), and transmitted 200x fewer data values than centralized coordination. Energy cost did not differ significantly from that of centralized coordination (*p* = 0.32), and the privacy-utility trade-off was negligible (0.016% cost variation). A direct noise-location experiment provided preliminary support at 4/8 noise levels: aggregation-stage noise is better tolerated than matched local-encoding noise, thereby supporting population-coding-inspired prediction in this cyber-physical testbed. Beyond the smart-grid application, these results support the broader computational principle that hierarchical distributed systems can maintain stable collective behavior when local influence is bounded, and stochastic perturbations are introduced at the aggregation layer. The proposed framework is not a biological model of a specific neural circuit and does not claim to train a deep neural network. Rather, it provides a reproducible cyber-physical testbed for computational-neuroscience questions about neural networks, including local error minimization, recurrent feedback, bounded unit influence, stochastic readout, and noise-tolerant consensus. This perspective can help connect privacy-preserving distributed optimization with theoretical models of large-scale neural coordination and motivate future studies on adaptive noise scheduling, graph neural coordination, and heterogeneous agent architectures inspired by cortical hierarchy models.

## Data Availability

Publicly available datasets were analyzed in this study. This data can be found here: the EirGrid demand and generation traces are publicly available at https://www.eirgrid.ie/grid/real-time-system-information and SEM-O historical market data at https://www.sem-o.com/market-data/static-reports/historic-market-data. All processed hourly traces, HEMS parameter files, simulation configuration files, and source code (Python/PyTorch) required to reproduce the experiments are deposited at https://github.com/AtefGharbi1/privacy-aware-federated-congestion-signal-coordination.
